# Early physiological and biochemical modulation in garlic under drought and high temperature

**DOI:** 10.3389/fpls.2026.1860343

**Published:** 2026-08-11

**Authors:** Tvrtko Karlo Kovačević, Nikola Major, Marina Krpan, Dean Ban, Anja Batel, Mario Franić, Smiljana Goreta Ban

**Affiliations:** 1 Department of Agriculture and Nutrition, Institute of Agriculture and Tourism, Poreč, Croatia; 2 Centre of Excellence for Biodiversity and Molecular Plant Breeding, Zagreb, Croatia; 3 Department of Nutrition, Quality and Food Safety, Faculty of Food Technology and Biotechnology, University of Zagreb, Zagreb, Croatia

**Keywords:** abiotic stress, adaptation, *Allium sativum*, chlorophyll fluorescence, metabolite profiling, multivariate analysis

## Abstract

Drought and high temperature are major abiotic stresses that increasingly threaten crop productivity under climate change. Although responses to individual stresses are widely documented, the combined effects of drought and heat often induce distinct physiological and metabolic adjustments that remain insufficiently understood. This study investigated short-term responses of garlic plants (cv. Istarski crveni) grown under water-available and ambient temperature (W+/T−), water-available and high temperature (W+/T+), drought-induced and ambient temperature (W−/T−), and drought-induced and high temperature (W−/T+) conditions in controlled climate chambers. Morphological and physiological traits (leaf color parameters, chlorophyll fluorescence, and dry matter), together with metabolite profile (abscisic acid, gamma-aminobutyric acid, proline, glycine, serine, glucose, fructose, mannitol), were assessed over an 8-day period using ANOVA and PLS-DA analyses. Exposure to drought reduced substrate water content and induced abscisic acid (ABA) accumulation, followed by increased proline levels and changes in leaf dry matter. When occurring with high temperature, accumulation of osmolytes, including glucose, fructose, and serine was observed, while photosystem II efficiency remained stable. Multivariate analysis revealed that temperature dominated treatment differentiation during first three days, whereas water availability became an important factor from day 4 onwards. Serine and fructose emerged as potential early biomarkers of combined drought and temperature stress, while leaf color and fluorescence parameters emerge as candidate non-destructive indicators of plant stress responses, warranting validation under field conditions.

## 1 Introduction

Plant survival and productivity are closely linked to the capacity of plants to perceive, respond, and adapt to a constantly changing environment (Zhu, 2016). Central to this ability is a finely tuned regulatory network driven by phytohormones (Santner et al., 2009; Verma et al., 2016). These compounds, though often present in minute concentrations, exert profound effects on plant physiology and development (Santner et al., 2009). The role of these molecules extends far beyond growth regulation, encompassing processes such as defense responses and integration of environmental signals (Verma et al., 2016). In particular, the dynamic interplay between hormonal signaling and plant morphology represents a crucial axis of control that sustains plant well-being across developmental stages and fluctuating environmental conditions (Tanaka et al., 2014).

Drought stress reduces turgor pressure, limiting cell expansion and division, resulting in smaller leaves, lower stomatal conductance, and reduced photosynthesis (Khamis et al., 2025; Zia et al., 2021). Prolonged water deficit induces osmolyte accumulation, such as proline and soluble sugars, for osmotic adjustment and protection, and triggers oxidative stress that activates antioxidant defenses (Hasanuzzaman et al., 2020; Oguz et al., 2022). Heat stress disrupts cellular processes and photosynthesis, causing protein denaturation, membrane instability, and increased respiration, while inducing the accumulation of osmolytes, amino acids, sugars, antioxidants, and heat shock proteins to maintain cellular homeostasis (Chen et al., 2023; Mondal et al., 2023). Combined drought and heat stress exacerbate negative impacts on growth and productivity, disrupting metabolism, reducing photosynthesis, impairing reproductive development, and causing greater yield losses than either stress alone (Suzuki et al., 2014).

Garlic (*Allium sativum* L.) is a globally cultivated crop with substantial nutritional, medicinal, and economic value (Kamenetsky et al., 2004). As climate change intensifies, marked by increasing temperatures and lack of rainfall, the demand for resilient, stress-tolerant cultivars is more urgent than ever (Fahad et al., 2017; Lesk et al., 2016). Garlic is sensitive to drought due to its shallow root system restricting the soil volume available for water uptake (Zhou et al., 2021), while high temperatures can increase respiration and nutrient consumption, excessive reactive oxygen species (ROS) production, leading to oxidative damage in plant tissues, all of which hinders subsequent growth and reduces both yield and quality (Yang et al., 2025). In garlic, drought is known to significantly affect leaf relative water content, chlorophyll concentration, and photosynthetic activity. At the same time, drought-increased dry matter and proline content point towards the importance of osmotic adjustment and redox homeostasis for drought tolerance (Habuš Jerčić et al., 2023; Kovačević et al., 2023). Drought-tolerant *Allium* genotypes (garlic and onion) also tend to accumulate higher levels of soluble sugars and fructans, together with free amino acids and enhanced antioxidant enzyme activities, underlining the roles of compatible solutes and ROS-scavenging systems in stress mitigation (Benkeblia, 2022; Gedam et al., 2021; Wei et al., 2024). High temperature stress in garlic and related *Allium* species is associated with membrane damage, altered ROS balance, reduced photosynthetic efficiency, and extensive changes in hormone signaling networks (Yang et al., 2025). Beyond single stresses, recent work indicates that combined drought and heat often causes stronger and more persistent reductions in photosynthesis and growth than either stress alone and triggers complex adjustments in osmolyte accumulation, phenylpropanoid metabolism, sulfur metabolism, and hormone crosstalk (Alhaithloul, 2019; Li et al., 2025; Zandalinas et al., 2022).

Although responses to individual stresses have been extensively studied, the combined effect of drought and heat often triggers distinct plant adaptations, particularly in biochemical networks, which remain poorly understood (Khan et al., 2025; Pandey et al., 2015). The present study investigates the effect of individual and co-occurring drought and high temperature in garlic plants, with the aim of elucidating underlying mechanisms of stress perception and tolerance, in addition to the identification of early stress biomarkers. The stress-responsive metabolites and physiological markers may represent candidate early diagnostic indicators and warrant further validation across cultivars, growing seasons, and field conditions, ideally linked to yield outcomes.

## 2 Materials and methods

### 2.1 Plant material and experimental design

The outermost cloves of the selected garlic (*Allium sativum* L.) cultivar Istarski crveni were provided from the gene bank collection of the Institute of Agriculture and Tourism, Poreč, Croatia, where the bulbs were stored under cool and dry conditions during the natural post-harvest dormancy period prior to planting. A total of 600 largest outermost cloves were planted (September 25^th^, 2023) in polystyrene containers containing an 8:5 mixture of peat and vermiculite. The plants were initially grown in a non-heated greenhouse at the Institute of Agriculture and Tourism in Poreč, Croatia (45°13′20.351″ N, 13°36′6.397″ E). During this time, garlic plants were watered according to standard agrotechnical practices, and the number of emerged plants was recorded. To reliably evaluate the effects of water deficit and high temperature, a two-factorial experiment was conducted in controlled climate chambers (GC-401, Nüve, Turkey). Thus, 40 days after planting, plants were transferred to two climate chambers for acclimatization under the following conditions: 16 h light/8 h dark photoperiod, day/night temperatures of 22°C/18°C, and 50% relative humidity (Bauer et al., 2022). At the onset of the experimental phase, one climate chamber was programmed to simulate high temperature (16 h light/8 h dark; 38°C day/24°C night; 50% relative humidity) using gradual temperature transitions at the start of each photoperiod to mimic natural diurnal warming, while the second chamber maintained the acclimatization conditions. At the same time, on half of the plants in each climate chamber, drought was imposed by withholding irrigation, and substrate moisture was monitored gravimetrically to maintain clearly differentiated water availability regimes. The selected single-factor stress intensities were based on relevant literature and our own preliminary optimization experiments, to ensure biologically meaningful and reproducible conditions. Overall, 4 treatment groups were established, each comprising 75 biological replicates: water-available and ambient temperature (W+/T−), water-available and high temperature (W+/T+), drought-induced and ambient temperature (W−/T−), and drought-induced and high temperature (W−/T+). During the experimental period, sampling was conducted every day by taking leaves of 7 plants per treatment.

### 2.2 Morphological and physiological measurements

Leaf color (L*, a*, b*) parameters were analyzed using a portable colorimeter (MiniScan EZ 4500, HunterLab, Reston, VA, USA), and chlorophyll fluorescence (F_0_, F_m_, F_v_/F_m_) and plants’ performance index (PI) were assessed using a Handy PEA+ fluorimeter (Hansatech, Pentney, UK) on 7 plants per sampling timepoint. Dry matter (DM) was determined according to ISO 11465:1993 by drying samples at 105°C in a forced-air oven (Memmert UF160, Schwabach, Germany) to constant weight in triplicate. DM was expressed as a percentage of dry weight in total fresh weight.

### 2.3 Substrate water content monitoring

**Figure 1:**
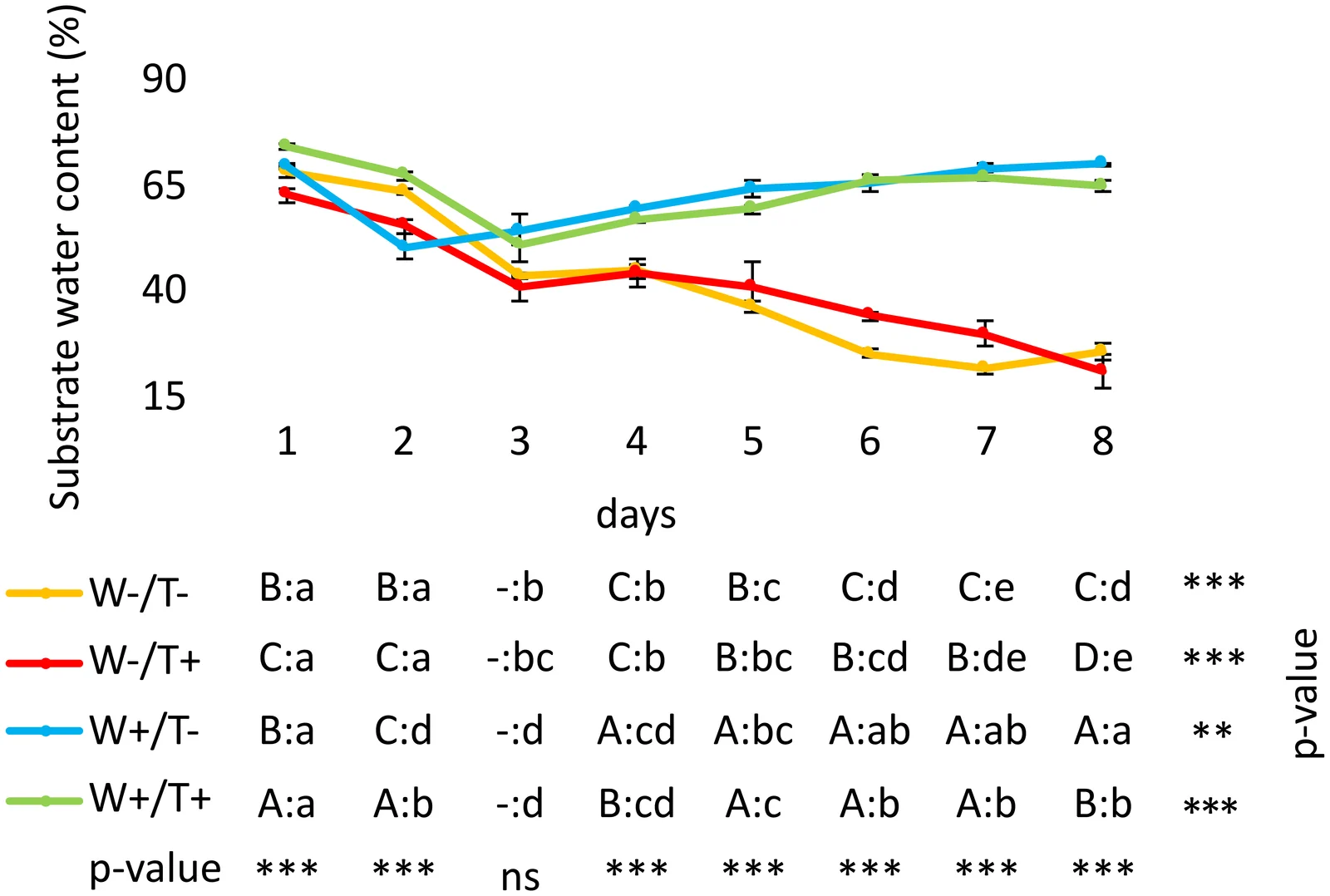
Substrate water content of garlic plants exposed to water-available and ambient temperature (W+/T-), water-available and high temperature (W+/T+), drought-induced and ambient temperature (W-/T-), and drought-induced and high temperature conditions (W-/T+). Capital letters indicate significant differences between treatments within days in Tukey’s *post-hoc* test, while small letters indicate significant differences between days within treatment groups in Tukey’s *post-hoc* test. ns, not significant; **p< 0.01; ***p< 0.001.

After plant sampling, the substrate from garlic plants exposed to water-available and ambient temperature (W+/T-), water-available and high temperature (W+/T+), drought-induced and ambient temperature (W-/T-), and drought-induced and high temperature conditions (W-/T+) were analyzed for water content by calculating the difference between the masses of the substrate before and after drying in a forced hot air circulation oven (Memmert UF160, Schwabach, Germany) held at 105°C according to ISO11465:1993 until constant weight (**Figure 1**). The results are expressed as mean ± standard error from 3 measurements.

### 2.4 Extraction of bioactive compounds

Young and healthy leaves (4 biological replicates per treatment group) were immediately flash-frozen in liquid nitrogen and lyophilized for 48 hours using a freeze dryer (Labogene ScanVac CoolSafe, Allerød, Denmark). Lyophilized samples were weighed (100 mg) and sequentially extracted in five steps using 1 mL of 80:20 methanol:water (v/v) at 30°C. Homogenization was performed using a bead mill homogenizer (Omni International, Kennesaw, GA, USA) at 6 m/s for 2 minutes. After each extraction step, samples were centrifuged at 16,000 × g for 10 minutes (Domel Centric 350, Železniki, Slovenia), and the supernatant was collected into a clean tube. Following the extraction, an aliquot was transferred into a clean Eppendorf tube and lyophilized (Labogene ScanVac CoolSafe, Allerød, Denmark), after which the sample was reconstructed in deionized water.

### 2.5 Metabolite identification and quantification

The metabolic profile was determined using an LC-MS/MS system, which included an autosampler (Shimadzu Nexera SIL-40CX3, Kyoto, Japan), two solvent delivery units (Shimadzu Nexera LC-40DX3, Kyoto, Japan), a thermostatic column compartment (Shimadzu Nexera CTO-40C, Kyoto, Japan), and a triple-quadrupole mass spectrometer (Shimadzu LCMS8045, Kyoto, Japan). Separation was carried out on a Discovery^®^ HS F5–3 column (2.1 mm × 150 mm, 3 µm core–shell, Sigma-Aldrich, St. Louis, MO, USA) at 37°C, according to Polić Pasković et al (Polić Pasković et al., 2024). A 1 µL sample was injected, and separation was achieved using a linear gradient of mobile phase A (water with 0.1% formic acid) and mobile phase B (acetonitrile with 0.1% formic acid) at a flow rate of 0.25 mL/min. The gradient program was as follows: 0–2 min: 100% A; 2–5 min: 100% A to 75% A; 5–11 min: 75% A to 65% A; 11–15 min: 65% A to 5% A; 15–20 min: 5% A; 20–20.1 min: 5% A to 100% A; and 20.1–25 min: 100% A. For sugar analysis, 1 µL of the sample was injected on a 4.6 x 250 mm Asahipak NH2P-50 4E column (Shodex, Tokyo, Japan) and sugars separated by a linear gradient of mobile phase A (water) and mobile phase B (acetonitrile) as follows: 0–20 min, 20%A to 50%A; 20-20.1 min: 50%A to 25%A; 20.1-25min: 25%A. The identification and quantification of targeted compounds were performed by comparing retention times, characteristic precursor/product ion pairs, and peak areas to reference standards. The results for total amino acids, total organic acids and total sugars are reported by summing the quantified metabolites into appropriate classes (**Supplementary Table 1**). The results for secondary metabolites were also assessed and presented in a companion study by the same authors (Kovačević et al., 2026).

### 2.6 Statistical analysis

The obtained data were tested for normality of residuals and homogeneity of variances using the Kolmogorov-Smirnov and Levene’s tests, respectively. A two-way analysis of variance (two-way ANOVA) was conducted to examine the effects of main factors, water-availability and temperature regime, along with their interaction on investigated variables. As interaction proved to be significant for each day, a one-way analysis of variance (one-way ANOVA) was conducted to investigate the differences between plants exposed to different conditions within days and between days within group. As homogeneity of variances was satisfied for all variables in the dataset, all variables were analyzed using the same statistical method, noting that ANOVA is generally robust to moderate deviations from normality, especially in balanced designs (n = 4 per group) (Schmider et al., 2010). To account for multiple testing across the daily one-way ANOVAs (344 comparisons in total), p-values were corrected for false discovery rate using the Benjamini-Hochberg procedure (Benjamini et al., 1995). Of the 241 comparisons significant at raw p< 0.05, 216 remained significant after correction (q< 0.05). For variables where significant differences were detected between plants exposed to different conditions within days and between days within group, Tukey’s HSD test was applied as a *post-hoc* analysis. Partial least squares discriminant analysis (PLS-DA) was further employed to identify the most important factors differentiating the treatment conditions (W+/T−, W+/T+, W−/T−, W−/T+) with quality parameters and variable importance in projection (VIP) values presented in **Supplementary Table 2**. All statistical analyses were conducted using XLSTAT (2007), a statistical software for Excel.

## 3 Results

### 3.1 Univariate analysis

**Figure 2:**
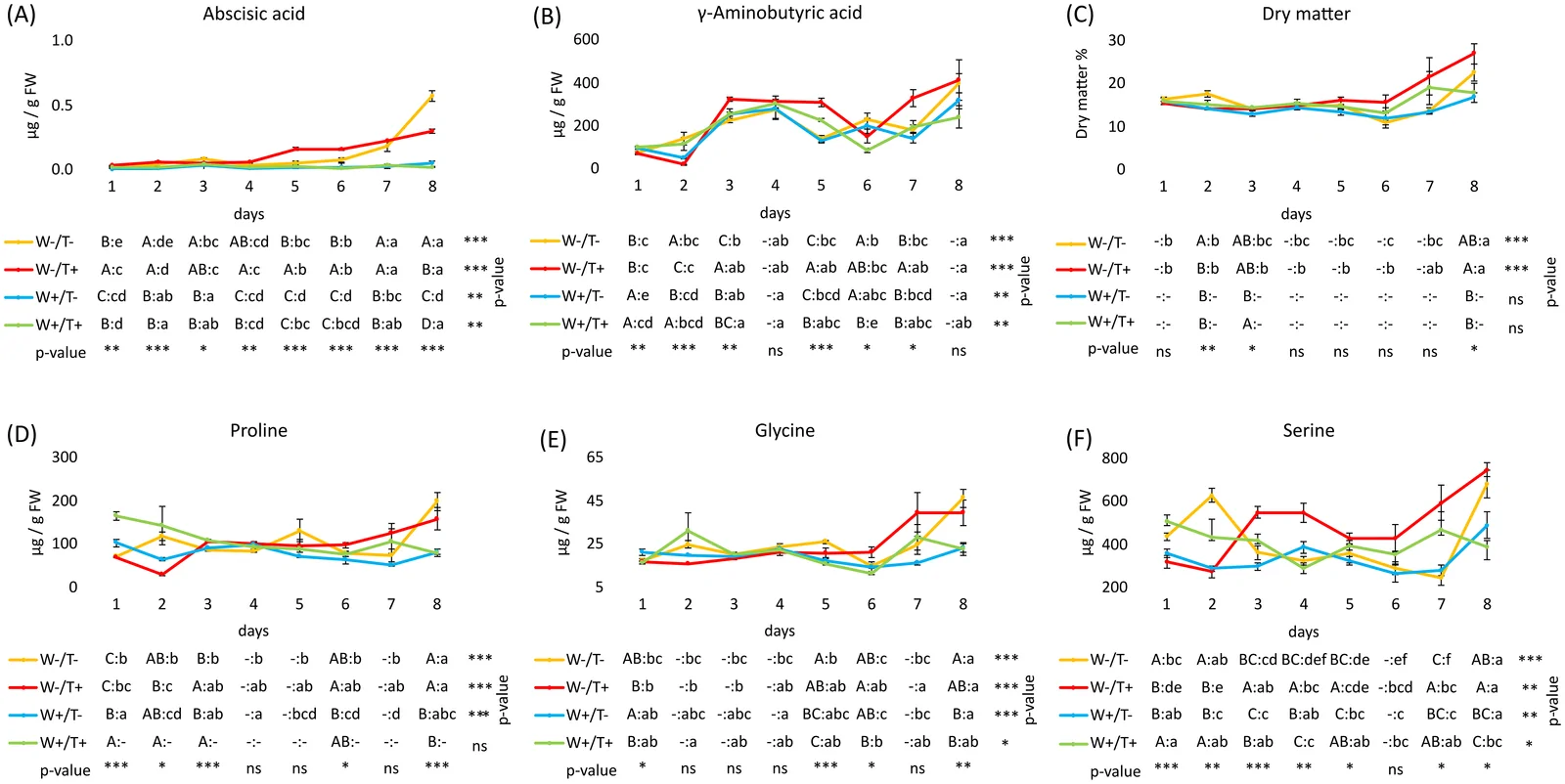
**(A)** Abscisic acid, **(B)** γ-Aminobutyric acid, **(C)** Dry matter, **(D)** Proline, **(E)** Glycine, and **(F)** Serine content of garlic plants exposed to water-available and ambient temperature (W+/T-), water-available and high temperature (W+/T+), drought-induced and ambient temperature (W-/T-), and drought-induced and high temperature conditions (W-/T+). Capital letters indicate significant differences between treatments within days in Tukey’s *post-hoc* test, while small letters indicate significant differences between days within treatment groups in Tukey’s *post-hoc* test. ns, not significant; *p< 0.05; **p< 0.01; ***p < 0.001.

During the first three days, garlic plants exposed to water-available and high temperature (W+/T+) exhibited significantly higher substrate water content compared to the remaining treatments, except on day 3 when no significant difference between the treatments was observed. From day 4 onwards, drought-induced (W-/T- and W-/T+) treatments exhibited significantly lower substrate water content compared to (W+/T- and W+/T+) treatments (**Figure 1**). As no clear effect of water deficit on substrate water content was evident during the first three days, significant difference in abscisic acid (ABA) leaf content during that period could also not be attributed to water deficit (**Figure 2A**). From day 5 onwards, the difference between treatment groups was evident, where plants exposed to drought (W-/T- and W-/T+) had significantly higher ABA content compared to watered plants (W+/T- and W+/T+), regardless of temperature (**Figure 2A**). Within drought-induced conditions, a significant increase in ABA content was observed after day 5 under high temperature (W-/T+), whereas at ambient temperature (W-/T-), the same was observed after day 7, compared to previous days (**Figure 2A**). Although differences between treatments in γ-aminobutyric acid (GABA) content were detected during the 8-day experiment, no clear effect of either temperature or water availability can be observed (**Figure 2B**). Nevertheless, leaf GABA content increased in all treatments by day 8 compared to the beginning of the experiment (**Figure 2B**).

Higher leaf DM was observed on day 8 in the drought/high temperature (W-/T+) plants compared to the watered plants (W+/T+ and W+/T- groups), while the drought-exposed plants at ambient temperature (W-/T-) were comparable in leaf DM (**Figure 2C**). By the end of the 8-day exposure period, leaf DM had significantly increased in plants exposed to drought (W-/T- and W-/T+) whereas no significant change in leaf DM was observed in watered plants (W+/T- and W+/T+) (**Figure 2C**).

**Figure 3:**
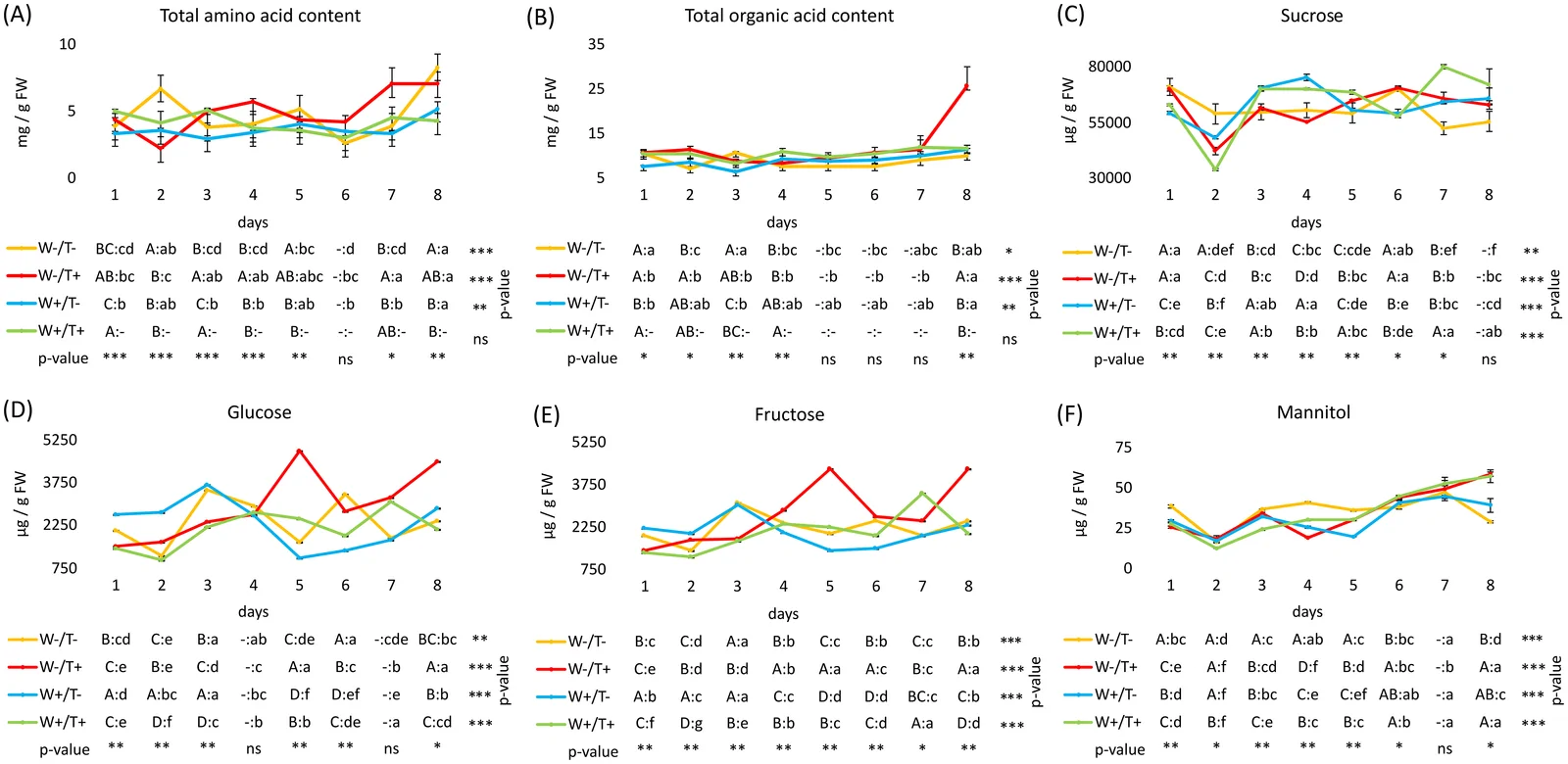
**(A)** Total amino acid content, **(B)** Total organic acid content, **(C)** Sucrose, **(D)** Glucose, **(E)** Fructose, and **(F)** Mannitol content of garlic plants exposed to water-available and ambient temperature (W+/T-), water-available and high temperature (W+/T+), drought-induced and ambient temperature (W-/T-), and drought-induced and high temperature conditions (W-/T+). Capital letters indicate significant differences between treatments within days in Tukey’s *post-hoc* test, while small letters indicate significant differences between days within treatment groups in Tukey’s *post-hoc* test. ns, not significant; * *p* < 0.05; ** *p* < 0.01; *** *p* < 0.001.

On day 8, plants exposed to drought (W-/T- and W-/T+) had significantly higher proline content compared to watered plants (W+/T- and W+/T+), reflecting the effects of lower substrate water and increased ABA content (**Figure 2D**). Proline content had significantly increased on day 8 compared to all previous days within W-/T- plants and compared to the first two days within W-/T+ plants, indicating a synergistic effect of water deficit and high temperature on proline content (**Figure 2D**). Differences between treatment groups in glycine content were observed on days 1, 5, 6, and 8, but no clear effect of water availability or temperature could be detected (**Figure 2E**). Glycine content within W-/T- plants had significantly increased on day 8 compared to the previous days. When plants were exposed to high temperature in addition to drought (W-/T+), higher glycine content was observed on days 7 and 8 compared to the first three days. Glycine content within watered plants (W+/T- and W+/T+) was comparable at the beginning and end of the experiment (**Figure 2E**). Significantly increased serine content was detected in W-/T+ plants compared to W+/T- plants from day 3 onward, except on day 6, when no significant differences between the treatment groups were observed (**Figure 2F**). Additionally, W-/T+ plants had significantly higher serine content compared to W+/T+ plants by day 8, which was not observed in plants at ambient temperature (W-/T- compared to W+/T-) (**Figure 2F**). As with the other investigated amino acids, serine content increased within plants exposed to drought (W-/T- and W-/T+) by day 8 compared to previous experimental days, except for day 2 for W-/T- plants and day 3 for W-/T+ plants. In watered treatments (W+/T- and W+/T+, respectively), the serine content by the end of the experiment remained comparable or even lower compared to day 1 (**Figure 2F**). Although significant differences between the treatments in total amino acid content during the experimental period were observed, no clear effect of water deficit could be detected, despite concurrent reduction in soil water content from day 4, and concurrent increase in ABA content from day 5 onwards in plants exposed to drought conditions (W-/T- and W-/T+) compared to watered plants (W+/T- and W+/T+) (**Figure 3A**). Total amino acid content in watered plants grown at high temperature (W+/T+) remained statistically unchanged throughout the experimental period, indicating that high temperature alone did not have a significant effect on the total amino acid content. Meanwhile, the remaining treatments had significantly higher total amino acid content by the end of the experiment, compared to day 1 of the experiment (**Figure 3A**).

The observed differences in total organic acid content among treatments during the first four days did not align consistently with either single or combined stress. Additionally, no significant differences were observed between the treatments in total organic acid content from day 5 to day 7 (**Figure 3B**). By day 8, plants exposed to drought and high temperature (W-/T+) had significantly higher total organic acid compared to the remaining treatments, and compared to previous experimental days, indicating a synergistic effect of water deficit and high temperature on total organic acid content (**Figure 3B**). On the other hand, the effect of single stress treatments of elevated temperature or drought was not reflected in organic acid content as these treatments were either comparable between day 3 and day 8 (for W-/T-) or not significant throughout the experiment (for W+/T+). Within W+/T- plants total organic acid content remained comparable between day 2 and day 8.

The most abundant sugar in garlic leaves was sucrose, accounting from 87.7% to 95.9% of all detected sugars. No clear effect of either drought or high temperature on sucrose content was observed, but at the end of experiment (day 8), plants exposed to drought, regardless of temperature (W-/T- and W-T+), had significantly lower sucrose content compared to the start of the experiment (day 1) (**Figure 3C**). The opposite trend, significant increase in sucrose content on day 8 compared to day 1, was observed in watered plants (W+/T- and W+/T+) (**Figure 3C**). The W-/T+ plants had significantly higher glucose and fructose content compared to all the other plants on day 8 (**Figures 3D, E**). Concurrent with significantly lower substrate water content from day 4 onwards, significantly higher glucose and fructose content was observed within W-/T+ plants from day 4 onwards compared to the first three days of the experiment (**Figures 3D, E**). The same effect was observed in leaf mannitol content but in W+/T+ plants (**Figure 3F**).

**Figure 4:**
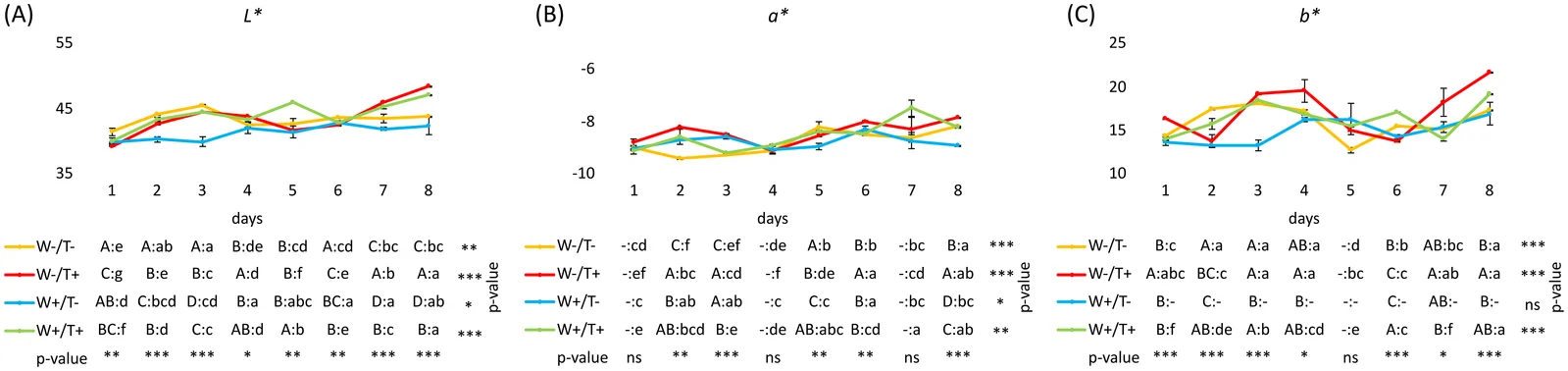
**(A)** Leaf lightness (L*), **(B)** greenness (a*), and **(C)** yellowness (b*) of garlic plants exposed to water-available and ambient temperature (W+/T-), water-available and high temperature (W+/T+), drought-induced and ambient temperature (W-/T-), and drought-induced and high temperature conditions (W-/T+). Capital letters indicate significant differences between treatments within days in Tukey’s *post-hoc* test, while small letters indicate significant differences between days within treatment groups in Tukey’s *post-hoc* test. ns, not significant; *p < 0.05; **p < 0.01; ***p < 0.001.

By days 7 and 8, both high temperature and drought significantly increased garlic leaf lightness (L*) (**Figure 4A**). Plants exposed to high temperature (W+/T+ and W-/T+) had significantly higher L* compared to those grown at ambient conditions (W+/T- and W-/T-), while drought-exposed plants (W-/T- and W-/T+) had significantly higher L* than watered plants (W+/T- and W+/T+) (**Figure 4A**). By day 8 of the experiment, the plants exposed to drought conditions (W-/T- and W-/T+) were characterized by decreased leaf greenness (a*) compared to watered plants (W+/T- and W+/T+) (**Figure 4B**). Also, the W-/T+ plants had significantly increased leaf yellowness (b*) compared to W-/T- and W+/T- plants (**Figure 4C**).

**Figure 5:**
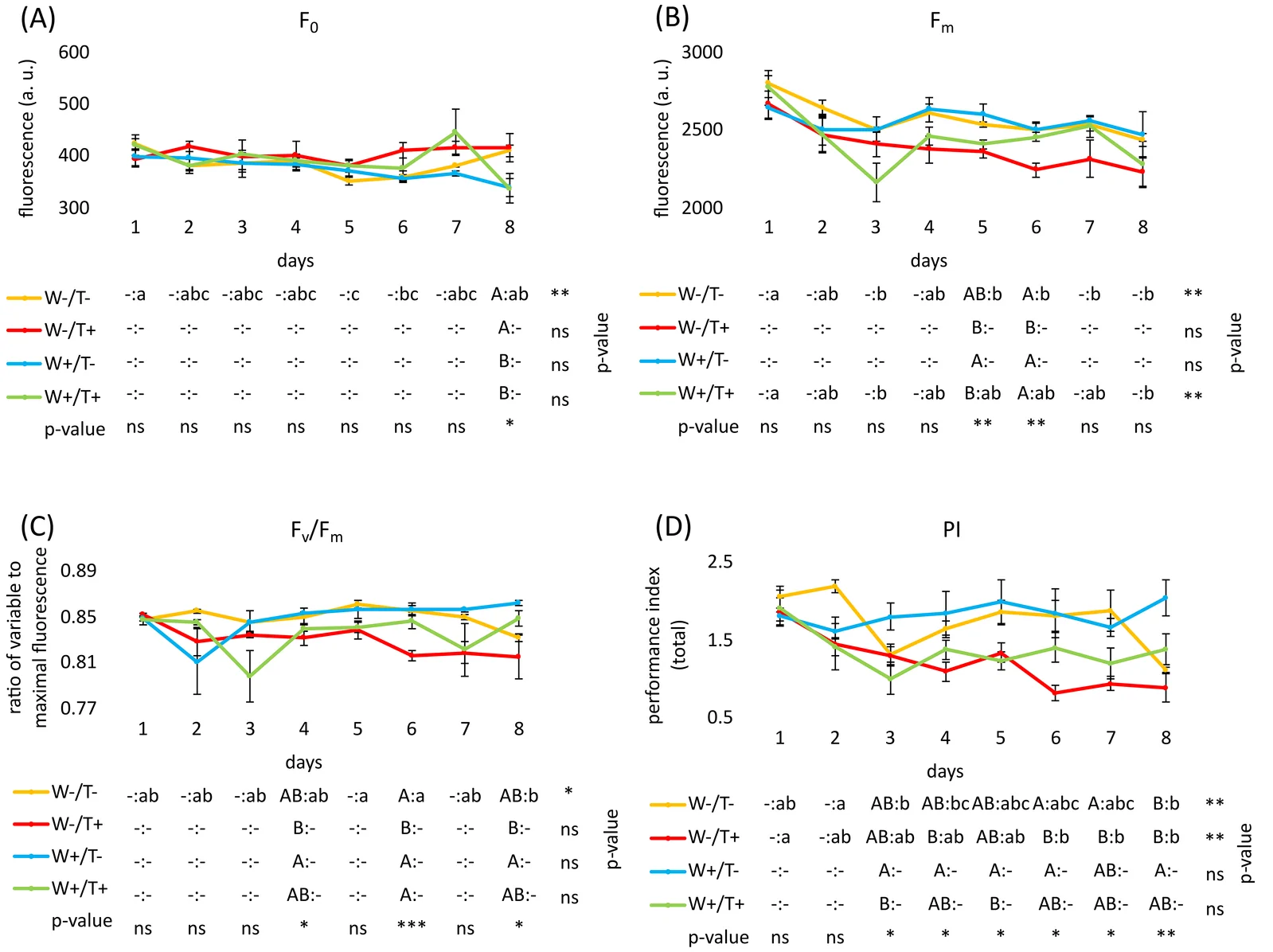
**(A)** Minimal fluorescence (F_0_), **(B)** Maximal fluorescence (F_m_), **(C)** Maximum quantum yield of PSII (F_v_/F_m_), and **(D)** Performance index (PI) measurements of garlic plants exposed to water-available and ambient temperature (W+/T-), water-available and high temperature (W+/T+), drought-induced and ambient temperature (W-/T-), and drought-induced and high temperature conditions (W-/T+). Capital letters indicate significant differences between treatments within days in Tukey’s *post-hoc* test, while small letters indicate significant differences between days within treatment groups in Tukey’s *post-hoc* test. ns, not significant; *p < 0.05; **p < 0.01; ***p < 0.001.

The photosynthesis parameters (F_m_, F_v_/F_m_, PI) did not show clear differences between the plants, except on day 8 when plants exposed to drought (W-/T- and W-/T+) had significantly higher minimum fluorescence (F_0_) compared to watered plants (W+/T- and W+/T+) (**Figures 5A–D**). Additionally, no clear differences within the treatments were observed, except for PI of plants exposed to simultaneous drought and high temperature (W-/T+), whose value by days 6, 7, and 8 had decreased compared to the days 1 and 2 (**Figure 5D**).

### 3.2 Multivariate analysis

**Figure 6:**
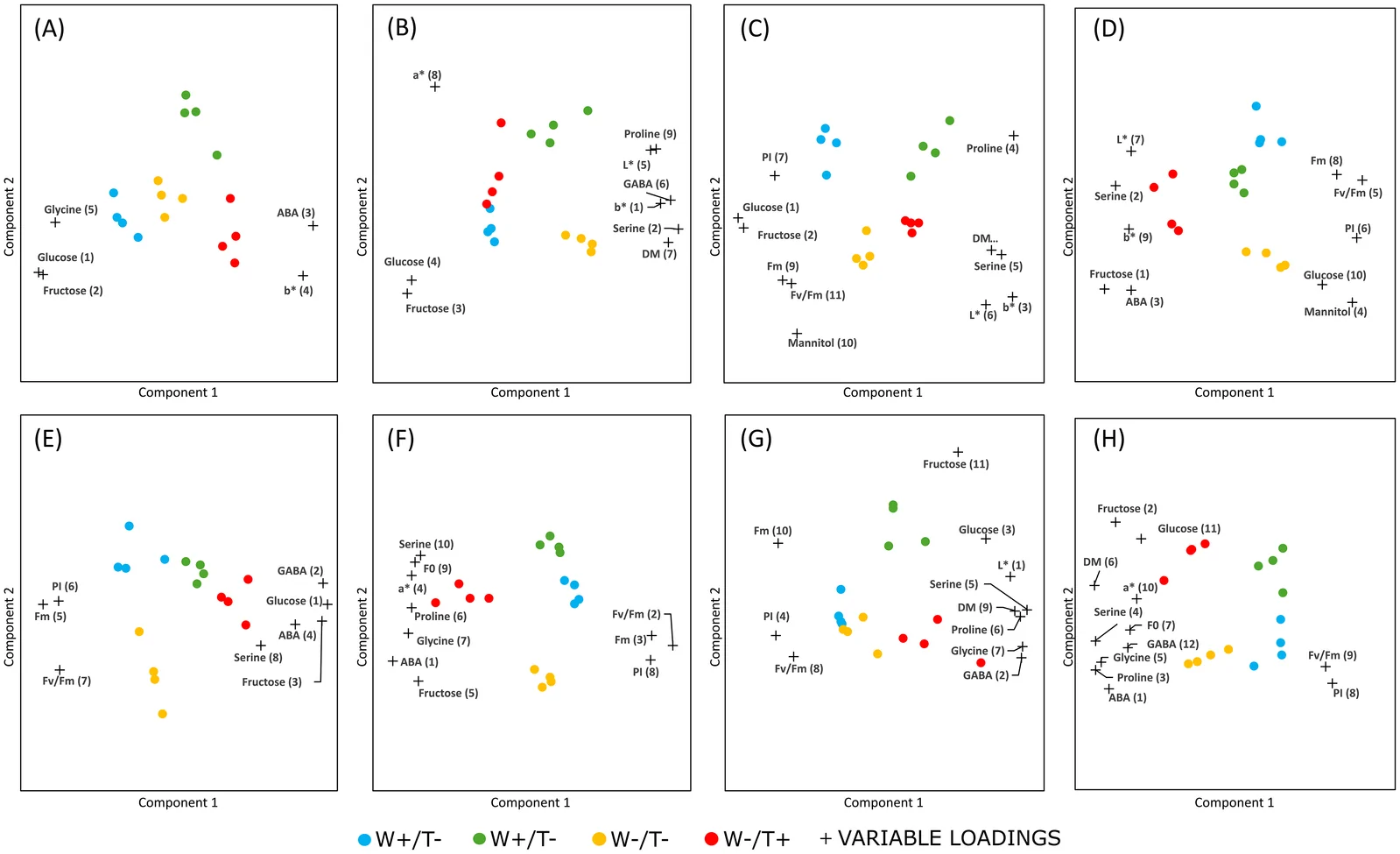
PLS-DA models for the differentiation between the garlic plants exposed to water-available and ambient temperature (W+/T-), water-available and high temperature (W+/T+), drought-induced and ambient temperature (W-/T-), and drought-induced and high temperature conditions (W-/T+) for day **(A)** 1, **(B)** 2, **(C)** 3, **(D)** 4, **(E)** 5, **(F)** 6, **(G)** 7, and **(H)** 8 of exposure. DM, leaf dry matter; F_0_, minimum fluorescence; F_m_, maximum fluorescence; F_v_/F_m_, maximum quantum yield of PSII; PI, performance index; L*, leaf lightness; a*, leaf greenness; b*, leaf yellowness; ABA, abscisic acid; GABA, γ-aminobutyric acid; W+/T-, water-available and ambient temperature conditions; W+/T+, water-available and high temperature conditions; W-/T-, drought-induced and ambient temperature conditions; W-/T+, drought-induced and high temperature conditions.

A multivariate statistical approach was employed to identify potential early stress markers in addition to the well-established stress-related compounds such as ABA and proline content. The PLS-DA models showed that during the first three days of the experiment, glucose and fructose content, as well as leaf yellowness were key parameters for the differentiation of plants grown at high temperature (T+) compared to plants grown at ambient temperature (T-) (**Figures 6A–C**). In addition, the PLS-DA model selected leaf lightness, proline and serine content on days 2 and 3 as important differentiating factors between the high and ambient temperature conditions (**Figures 6B, C**). The first differentiation of the treatment groups according to water availability was shown on the model for day 3, but as the second source of variation between the data, after temperature (**Figure 6C**).

On days 4, 5, and 6, the PLS-DA models showed that the plants exposed to simultaneous drought and high temperature (W-/T+) were clearly differentiated from the remaining treatment groups on the first component, while the W-/T- plants were separated from the W+/T- and W+/T+ plants on the second component (**Figures 6D–F**). The models identified ABA content as the main and fructose content as the co-occurring parameter in the differentiation between plants exposed to drought (W-) compared to watered plants (W+) (**Figures 6D–F**). The models also selected serine content as an important parameter, further separating plants exposed to simultaneous drought and high temperature compared to the other investigated groups (**Figure 6D**). During this period, the models also identified additional parameters contributing to group separation, including leaf lightness and yellowness on day 4, glucose and GABA content on day 5, and proline and glycine content on day 6. (**Figures 6D–F**).

On day 7, the model identified leaf lightness as the most important factor contributing to the separation between the treatment groups (**Figure 6G**). Under simultaneous drought and high temperature, the model identified leaf dry matter as a parameter contributing to group discrimination (**Figure 6G**). The developed model for the last day of the experiment showed clear differentiation of treatment groups according to water availability and temperature (**Figure 6H**). The strongest effect on group discrimination along component 1 was according to water availability, whereas group discrimination along component 2 was according to temperature. The model identified ABA, GABA, proline, and glycine content as the main parameters differentiating plants exposed to drought (W −) from watered plants (W+), irrespective of temperature. Under drought conditions, high temperature further contributed to the separation from the other treatment groups, with glucose, fructose, and serine content, as well as leaf greenness and dry matter, identified as the key discriminating parameters (**Figure 6H**).

The predictive performance of the daily PLS-DA models varied (cumulative Q² = 0.16-0.47), being strongest for the mid-experiment days (days 4-7) and weakest on days 1 and 3. The earliest-day separations should therefore be interpreted as exploratory, whereas the serine- and fructose-based discrimination of combined stress rests on the better-validated models.

## 4 Discussion

### 4.1 Univariate analysis of treatment effects (ANOVA)

Drought and high temperature are critical abiotic factors limiting crop productivity by impairing resource allocation, biomass growth and development, photosynthetic efficiency and leaf pigmentation (Hu F. et al., 2023; Kopecká et al., 2023). When co-occurring, drought and high temperature exert stronger effects, markedly decreasing chlorophyll content and photosynthetic efficiency compared to individual stresses and consequently impairing plants’ productivity (Alhaithloul, 2019; Hussain et al., 2019). Either way, these stresses elicit adjustments in plant metabolic profiles, including sugars, polyols and amino acids to stabilize proteins and membranes and to maintain osmotic and redox homeostasis (Renzetti et al., 2024), while signaling molecules such as ABA and GABA can accumulate rapidly, coordinating stress responses such as stomatal closure, osmotic adjustment, and protection of the photosynthetic apparatus (dos Santos et al., 2022; Gao et al., 2025).

In our experiment, the substrate water content decreased from day 4 onwards on plants exposed to drought (W-/T- and W-/T+), which lead to significantly increased production of ABA as a stress signaling molecule from day 5 onwards, compared to watered plants (W+/T- and W+/T+) (**Figures 1**, **2A**). In addition, high temperature in conjunction with drought enhanced ABA biosynthesis during days 5 and 6, and altered responses by day 8, indicate temperature-dependent modulation of response intensity and timing (Huang et al., 2016). Similarly to Jensen et al. (2024), increased ABA biosynthesis in our study was primarily triggered by drought, while temperature influenced the magnitude of the response, suggesting that plant water status acted as the principal inductive signal with temperature exerting a secondary modulatory effect. ABA is a well-established critical plant hormone having a major role in plant responses to stress (Latha et al., 2023; Rehman et al., 2022). Increased biosynthesis of ABA in plants exposed to drought has been widely reported (Zhou et al., 2021) across different plants species, such as in garlic leaves (Liu et al., 2023), eucalyptus shoots (Correia et al., 2018), wheat (Itam et al., 2020), and tomato (Jensen et al., 2024). Since ABA mediates so many stress responses, the initial perception of dehydration and the subsequent changes in gene expression that lead to rapid ABA biosynthesis constitute the most important stress signal transduction pathway among all the plant responses to stresses (Zhang et al., 2006). Our results showed that exposure only to high temperature was not sufficient to trigger ABA biosynthesis as W+/T+ plants showed no significant changes in ABA content compared to the W+/T- plants from day 4 onward, with the exception of day 8 (**Figure 2A**). Similar results have been reported across different plant species, where ABA levels remained stable under high temperature conditions, such as in eucalyptus (Correia et al., 2018), tomato (Jensen et al., 2024), and reed plants (Ding et al., 2010).

Reduced water availability enhances ABA biosynthesis, which activates ABA-dependent pathways that promote the accumulation of osmoprotective metabolites, including proline, thereby facilitating osmotic adjustment and sustaining cellular turgor under water-deficit conditions (Verslues and Sharma, 2010). Increased proline biosynthesis and accumulation is widely recognized as an early and sensitive indicator of abiotic stress, rapidly increasing in plant tissues in response to drought and osmotic imbalance and serving both protective and signaling functions in stress acclimation (Renzetti et al., 2024; Trovato et al., 2021). Increased content of proline was evident by day 8 in plants exposed to drought, regardless of temperature (W-/T- and W-/T+) compared to watered plants (W+/T- and W+/T+) (**Figure 2D**), consistent with findings across different plants species, such as in garlic leaves (Habuš Jerčić et al., 2023; Kovačević et al., 2023), eucalyptus (Correia et al., 2018), tobacco (Demirevska et al., 2010), peach leaves (Haider et al., 2018), and wheat (Guo et al., 2018).

However, studies show that proline responses to high temperature can vary widely among plant species (Szabados and Savouré, 2010), with some species, such as tomato leaves (Rivero et al., 2004), showing increased proline content, while others, such as moth bean (Harsh et al., 2016) exhibit reduced or no significant changes, highlighting species-specific differences in proline metabolism (Kavi Kishor et al., 2022). In addition, the severity and duration of high temperature exposure are key factors determining the magnitude and direction of proline accumulation in plants (Hasanuzzaman et al., 2013). Our results showed no significant differences in proline content in W+/T+ and W-/T+ compared to W+/T- and W-/T- plants, except for day 3, which suggest that high temperature has a weak effect on early proline accumulation in garlic compared to drought (**Figure 2D**).

In addition to ABA-dependent signaling pathway, drought-enhanced ABA biosynthesis also influences the sugar-mediated signaling pathway which in turn promotes biosynthesis and accumulation of soluble sugars and sugar alcohols (sucrose, glucose, fructose, and mannitol) (Eveland and Jackson, 2012). However, in the present study the described pattern was not clearly reflected in sucrose and glucose contents of plants exposed to drought (W-/T-) compared to watered plants (W+/T-), while it was evident in fructose content, as it increased from day 3 onwards (except for day 7) in former compared to latter. Similarly, mannitol content of plants exposed to drought (W-/T-) was higher compared to watered plants (W+/T-) for the first five days (except for day 2) (**Figures 3C–F**). In addition, Habuš Jerčić et al (Habuš Jerčić et al., 2023). reported no significant differences in sucrose and glucose contents between drought-induced and watered plants in garlic leaves of the same cultivar (cv. Istarski crveni), with a similar pattern observed in the present study. Studies by Kaplan et al (Kaplan et al., 2004). and Wahid et al (Wahid et al., 2007). show that high temperature alters carbon metabolism, transiently increasing the contents of soluble sugars, while prolonged stress can reduce carbohydrate pools. This short-lived rise in sugar content under higher temperature was reflected in the present study as well, as W+/T+ plants had higher sucrose content on days 5 and 7, higher glucose on days 5 and 6, higher fructose from day 4 up to day 7, and higher mannitol content on days 4 and 5, compared to W+/T- plants (**Figures 3C–F**). When occurring simultaneously with drought, high temperature may further reduce tissue water content due to increased evaporative demand and transpiration rates, thereby intensifying the need for the synthesis and accumulation of soluble sugars and sugar alcohols (Wahid et al., 2007). This pattern was evident for glucose, fructose, and mannitol, but not for sucrose content (**Figures 3C–F**). Glucose and fructose contents of W−/T+ plants increased significantly towards day 8 compared to W+/T- plants, as well as compared to all the previous days, except for day 5 (**Figures 3D, E**). Similarly, mannitol content increased in W−/T+ plants by day 8 compared to previous days (**Figure 3F**). In contrast, sucrose did not show the same response, as no clear effect of either drought or high temperature was observed during the experimental period (**Figure 3C**). Therefore, the carbohydrate response under simultaneous drought and high temperature was not characterized by a uniform increase in all soluble carbohydrates, but rather by selective and time-dependent accumulation of specific compounds, mainly glucose, fructose, and mannitol. This supports the interpretation that combined stress can induce a compound-specific metabolic adjustment rather than a direct treatment effect increase in carbohydrate content (Suzuki et al., 2014).

Although differences in GABA content between treatments were detected during the 8-day experiment, consistent effect of either temperature or water availability was not observed (**Figure 2B**). This may suggest that changes in GABA content are not connected to early stress response in garlic but may emerge under prolonged stress exposure as reported in literature (Kinnersley and Turano, 2000). To support this, Bor et al. (2009) reported higher GABA levels in sesame leaves after 21 days of drought compared with those measured after 7 days of stress exposure (Bor et al., 2009).

Similar to GABA, significant differences in glycine content between treatments were observed during the experimental period, but consistent effect of water availability or temperature was not observed (**Figure 2E**). Nonetheless, glycine content of W-/T- and W-/T+ plants had increased significantly from the beginning to the end of the experiment, whereas in watered plants (W+/T− and W+/T+) it remained comparable between the beginning and the end of experiment (**Figure 2E**). In the photorespiratory pathway, the conversion of two molecules of glycine into one molecule of serine is often cited as a major metabolic bottleneck (GDC bottleneck), particularly under stress conditions such as drought and high temperatures, which increase the oxygenase activity of Rubisco (Hodges et al., 2016). Thus, if photorespiration was the predominant mechanism, we would expect to observe a concurrent and disproportionate increase in glycine of stressed plants due to the GDC bottleneck. The absence of this glycine accumulation suggests that the observed serine increase is derived from an alternative source, such as phosphorylated pathway (Igamberdiev and Kleczkowski, 2018). Consistent with that, increased serine content was observed in W−/T+ plants compared to W+/T− plants from day 3 onward, except on day 6, when no significant differences between the treatments were detected (**Figure 2F**).

Consistent with the significantly lower substrate water content in plants exposed to drought (W−/T− and W−/T+) from day 4 onward (**Figure 1**), leaf DM increased significantly in these treatments by day 8 (**Figure 2C**). The higher leaf DM of W−/T+ plants compared to W+/T- and W+/T+ plants on day 8 may partly reflect reduced tissue water content (Chaves et al., 2003) (**Figure 2C**). However, the concurrent accumulation of metabolites such as proline, glycine, serine, glucose, fructose, and mannitol in W-/T+ plants suggests that active metabolic adjustment also contributed to the observed increase in leaf DM, pointing to a synergistic effect of simultaneous drought and high temperature (Farooq et al., 2009).

Drought and high temperature are known to impair photosynthetic activity by disrupting photosystem II (PSII), often increasing F_0_ while reducing F_m_, F_v_/F_m_, and PI, collectively reflecting reduced photosynthetic efficiency under abiotic stress (Kalaji et al., 2016). In the present study, F_0_ was significantly higher on day 8 in plants exposed to drought (W−/T− and W−/T+) compared to watered plants (W+/T− and W+/T+). However, the F_v_/F_m_ values in drought-exposed plants (W−/T− and W−/T+) remained within the range typical for non-stressed plants (~0.80), indicating that PSII maintained functional stability, while the increase in F_0_ likely reflects minor adjustments in PSII energy transfer rather than substantial impairment of photochemical efficiency (Baker, 2008). This overall stability may reflect the inherent drought tolerance of cultivar Istarski crveni, as previously reported by Kovačević et al. (2023), even under high temperature.

Taken together, the total amino acid, organic acid, and sugar contents, along with chlorophyll fluorescence parameters, suggest that biochemical adjustments preceded or accompanied the early effects of drought stress on photosynthetic performance. In the present study, most fluorescence parameters, including F_m_, F_v_/F_m_, and PI, did not show clear treatment-dependent differences during the experimental period, indicating that PSII efficiency was largely maintained under the imposed conditions. However, the significant increase in F_0_ in drought-exposed plants (W-/T- and W-/T+) on day 8 suggests that reduced water availability began to affect the photosynthetic apparatus toward the end of the experiment. This coincided with increased total amino acid content in drought-exposed plants, supporting the view that amino acid accumulation was part of drought-associated metabolic adjustment, including osmotic regulation, redox balance, and nitrogen redistribution (Živanović et al., 2020). Similarly, the increase in total organic acid content under simultaneous drought and high temperature conditions (W−/T+) on day 8 corresponded with the decline in PI observed in the same treatment from days 6 to 8. Although this may indicate an early adjustment of central carbon metabolism under simultaneous drought and high temperature stress, the stable F_v_/F_m_ values suggest that the photosynthetic apparatus was not severely impaired during the experimental period. Therefore, the observed changes in total organic acid content should be interpreted cautiously as part of a short-term metabolic adjustment rather than as evidence of pronounced disruption of photosynthetic performance or energy metabolism (de Oliveira et al., 2025). Furthermore, the absence of significant differences in sucrose content between treatments at the end of the experimental period, coupled with preserved F_v_/F_m_ values, indicates that short-term drought and high temperature did not cause a generalized collapse of photosynthetic function (Xu and Fu, 2022). Instead, the carbohydrate response appeared more compound-specific, as reflected in changes in glucose, fructose and mannitol rather than in the total quantified sugar pool, consistent with reports that soluble sugar responses to simultaneous heat and drought can be transient and stress-duration dependent (Zinta et al., 2018).

Despite lack of clear differences in photosynthetic parameters (except F_0_ on day 8), plants exposed to simultaneous drought and high temperature (W−/T+) had significantly higher leaf lightness and yellowness and reduced greenness compared to W+/T− plants, consistent with stress-induced chlorophyll loss and associated color shifts toward less greener, lighter and more yellow-brown hues (Hu W. et al., 2023; Nunta et al., 2023). In addition, leaf colorimetry revealed that the plants exposed to simultaneous drought and high temperature (W-/T+) had a significantly higher leaf lightness compared to both the watered plants (W+/T-) and single-stress treatments (W-/T- and W+/T+). While individual heat or drought stress initiates pigment breakdown, their co-occurrence synergistically accelerates cellular dehydration and the degradation of light-absorbing matrices, thus maximizing tissue reflectance, rendering the leaves significantly more pallid (Abdelhakim et al., 2021).

### 4.2 Multivariate approach to early stress marker discovery

According to the PLS-DA models, temperature was one of the sources of data variation during the first three days of the experiment (**Figures 6A–C**) with glucose and fructose contents as the most important variables in this separation. Additionally, proline and serine contents were also identified as important variables in the differentiation between ambient and high temperature conditions by day 3. The available literature showed that during the initiation of heat stress, plants were observed to decrease in sugar (Hong et al., 2023) and increase in amino acids such as proline (Renzetti et al., 2024) and serine (Kishor et al., 2020). The developed model also showed the emergence of water availability as a secondary source of variation on day 3 (**Figure 6C**) with increased leaf lightness as important variable distinguishing drought-exposed (W−) from watered plants (W+). Exposure to drought has been shown to affect chlorophyll and carotenoid contents, as well as their ratio, which can be perceived as changes in leaf lightness and/or color (Filimon et al., 2016).

ABA acts as a systemic signal that triggers the synthesis and accumulation of protective osmolytes, including soluble carbohydrates and various amino acids, to facilitate osmotic adjustment and maintain cellular turgor when plants are exposed to unfavorable environmental conditions (Sah et al., 2016; Vishwakarma et al., 2017). Thus, ABA is well-documented as an early stress marker (Cutler et al., 2010), and was also identified by our PLS-DA models from day 4 to day 6 as a highly important differentiating factor between watered and drought-induced conditions, coinciding with the progressive decline in substrate water content under drought. The models also identified higher fructose and serine contents as key parameters distinguishing plants exposed to simultaneous drought and high temperature (W-/T+) from the remaining treatments along component 1 (**Figures 6D–F**) highlighting these compounds as early stress markers for simultaneously occurring drought and high temperature.

Such differentiation between W-/T+ plants and the remaining treatments along component 1 was observed on day 7 as well, with glucose, serine, proline, glycine, and GABA, together with leaf lightness and DM, being identified by the model as the most influential variables driving this differentiation (**Figure 6G**). By day 8, water availability became the primary source of data variation, as the model identified F_0_, leaf DM, leaf greenness, ABA, proline, serine, glycine, and GABA as the most important variables distinguishing drought-exposed (W−) and watered plants (W+) along component 1 (**Figure 6H**). Meanwhile, temperature represented the secondary source of variation along component 2, with glucose and fructose contents being identified by the model as important variables contributing towards differentiation between W-T+ and W-/T- plants (**Figure 6H**).

In addition to well-established drought stress markers such as ABA and proline, the present PLS-DA results suggest that fructose and serine may also serve as early indicators of stress, particularly under simultaneous drought and high temperature (W−/T+). This synergistic accumulation of serine (Zandalinas et al., 2018) further corroborates its role during osmotic stress, where it may represent an unique metabolic fingerprint, distinct from the response to individual stressors (Obata et al., 2015; Rizhsky et al., 2004). Leaf DM may serve as a secondary marker reflecting experimental conditions. However, quantification of serine, fructose, and DM requires destructive sampling. Thus, leaf color (lightness, yellowness, and greenness) together with fluorescence parameters (F_m_, F_v_/F_m_, and PI) present a promising, non-destructive basis for future field-level monitoring of stress responses in garlic, pending validation in multi-cultivar, multi-season field trials that also assess yield impact. Together, these findings contribute to a better understanding of garlic stress physiology and may support the development of improved strategies for monitoring and enhancing crop resilience under increasingly frequent drought and heat events.

## 5 Limitations

A limitation of this study is that each temperature regime was applied in a single climate chamber (one chamber for ambient, one for high temperature), so chamber-specific effects are structurally confounded with the temperature treatment itself and cannot be statistically disentangled from it. To reduce the risk of positional artefacts within each chamber, plants were rotated daily, and actual temperature, humidity, and light conditions were continuously logged and confirmed to match the programmed set points throughout the experiment.

## Data Availability

The raw data supporting the conclusions of this article will be made available by the authors, without undue reservation.
